# An Experimental Study of Membrane Contactor Modules for Recovering Cyanide through a Gas Membrane Process

**DOI:** 10.3390/membranes10050105

**Published:** 2020-05-19

**Authors:** Michelle Quilaqueo, Gabriel Seriche, Sicely Valetto, Lorena Barros, Simón Díaz-Quezada, René Ruby-Figueroa, Elizabeth Troncoso, Humberto Estay

**Affiliations:** 1 Advanced Mining Technology Center (AMTC), University of Chile, Av. Tupper 2007 (AMTC Building), Santiago 8370451, Chile; michelle.quilaqueo@amtc.cl (M.Q.); gabriel.seriche@amtc.cl (G.S.); lorena.barros@amtc.cl (L.B.); simon.diaz@amtc.cl (S.D.-Q.); 2 Department of Chemical Engineering, Biotechnology and Materials, University of Chile, Av. Beauchef 851, Santiago 8370456, Chile; sicely.valetto@ing.uchile.cl; 3 Programa Institucional de Fomento a la Investigación, Desarrollo e Innovación, Universidad Tecnológica Metropolitana, Ignacio Valdivieso 2409, San Joaquín, Santiago 8940577, Chile; rruby@utem.cl (R.R.-F.); elizabeth.troncoso@utem.cl (E.T.); 4 Department of Chemistry, Universidad Tecnológica Metropolitana, Las Palmeras 3360, Ñuñoa, Santiago 7800003, Chile

**Keywords:** gas membrane process, cyanide recovery, scaling-up, membrane modules

## Abstract

Cyanide is one of the main reagents used in gold mining that can be recovered to reduce operational costs. Gas membrane technology is an attractive method for intensifying both the stripping and absorption processes of valuable compounds, such as cyanide. However, scaling-up this technology from laboratory to industry is an unsolved challenge because it requires the improvement of the experimental methodologies that replicate lab-scale results at a larger scale. With this purpose in mind, this study compares the performance of three different hollow fiber membrane contactor modules (1.7 × 5.5 Mini Module, 1.7 × 10 Mini Module, and 2.5 × 8 Extra Flow). These are used for recovering cyanide from aqueous solutions at laboratory scale, using identical operational conditions. For each experimental set-up, mass-transfer correlations at the ranges of feed flows assayed were determined. The modules with the smallest and largest area of mass transfer reached similar cyanide recoveries (>95% at 60 min), which demonstrate the impact of module configuration on their operating performance. The results obtained here are limited for scaling-up the membrane module performance only because operating modules with the largest area results in a low *Re* number. This fact limits the extrapolation of results from the mass-transfer correlation.

## 1. Introduction

The gas membrane process or gas-filled membrane absorption (GFMA) process has been widely studied for recovering or removing volatile elements contained in different sources. Recently, the GFMA process has been used to recover ammonia [1], CO_2_ [2,3], H_2_S [4], HCN [5] or to separate CH_4_ and CO_2_ [6]. The interest in this process resides in the high specific area (contact area per equipment volume) provided by membrane contactor modules [7], and the possibility to conduct the stripping and absorption steps in the same equipment in comparison with traditional equipment (packed beds, spray columns, bubble columns, venturi scrubbers, etc.). Here, these steps are undertaken separately. These advantages have promoted the study of this technology for cyanide recovery in gold mining [8,9,10,11,12] as an alternative to the conventional method based on packed towers for both stripping and absorption stages, namely the AVR (acidification, volatilization and recycling) process [13]. In fact, the GFMA process has an additional advantage with respect to the AVR technology, related to safety management. In the AVR process, there is a gas-flow of air containing HCN which forces the inclusion of a complex and redundant control system. By contrast, the GFMA process encloses the gaseous HCN inside the pores of the membrane, limiting the risks of HCN leaking [8,10,12].

Notwithstanding the advantages of the GFMA process, this technology is still far from ready for application at industrial-scale cyanide recovery in gold mining. This is because scaling-up results obtained from the laboratory scale towards an industrial case lack an experimental and theoretical methodology. Possible causes are: the high quantity of types of membrane contactor modules and their respective mass-transfer correlations [11], differences between the traditional industrial modules available and modules used at laboratory scale [12], and variability of the real solution characteristics. In a previous work, a methodology to scale-up the GFMA process, based on a phenomenological model, was developed [12]. This study proposed that the mass-transfer coefficient must be estimated to obtain the overall mass-transfer coefficient. This value can be obtained at laboratory scale and then used to be fixed to scale-up at pilot or industrial scale, using the proper mass-transfer correlation of a specific module. This study included economic aspects and the inclusion of different types of modules and operational conditions. However, the methodology used by the previous study depends on the specific mass-transfer correlation associated with each type of module. It is known that the mass-transfer correlation depends on the specific system (e.g., solutes and solvents) and operational conditions used to determine it. Therefore, the assessment of different types of modules at laboratory scale for each specific system is necessary to give support to any methodology to scale-up and design an industrial plant. Under this scenario, this study aims to compare the performance of three different types of membrane contactor used to recover cyanide in the GFMA process, at laboratory scale, operated in the same conditions. Thus, the results of different membrane module configurations could allow mass-transfer correlations and mass-transfer coefficients to be obtained for scaling-up its performance into membrane modules of larger scale. In this study we propose an experimental methodology able to relate the results obtained from typical modules used at laboratory scale with those operated at industrial scale. This methodology is based on a system operating at similar feed-flow ranges in order to fix the same experimental scale for each module. This work intends to move forward the industrial application of this technology in gold mining.

## 2. Materials and Methods 

### 2.1. Experimental Set-Up

Following the methodology previously used by Estay et al., (2013), synthetic cyanide and absorption solutions were prepared. A synthetic cyanide solution of concentration 2500 mg/L total CN^-^ was prepared using NaCN (99% purity) (Sigma-Aldrich, St. Louis MO, USA), and demineralized water (~5 μS), whose pH was adjusted to 3.5, using sulfuric acid of 95% *w*/*w* (Sigma-Aldrich). The absorption solution was prepared with NaOH (99% purity) (Sigma-Aldrich), and demineralized water (~5 μS), with concentration of 5% *w*/*w* NaOH.

**Figure 1:**
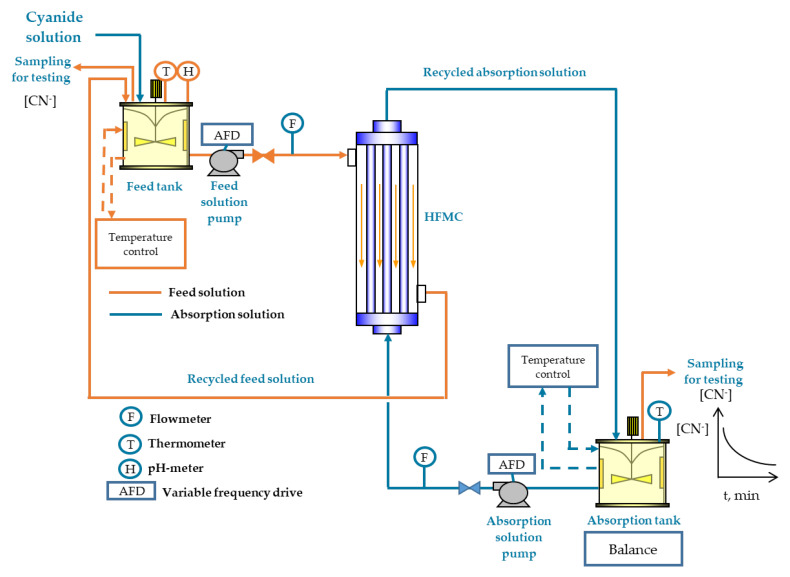
Laboratory scale experimental set-up for the gas-filled membrane absorption (GFMA) process.

The experimental set-up of the GFMA process at laboratory scale was composed of two jacketed, stirred, and sealed tanks with a capacity of 2 L, two peristaltic pumps (Masterflex, Model 77913-70, Gelsenkirchen, Germany) and a hollow-fiber membrane contactor (HFMC, described in Section 2.2) module (Figure 1). The cyanide solution was fed into the HFMC module through the shell side, and the absorption solution (5% *w*/*w* NaOH) was circulated in the lumen side in a countercurrent configuration. Both solutions were fed into the module at 15 °C. These solutions were permanently recycled into the respective tank. The pH was measured by a pH meter (Methrom, Model 913, Herisau, Switzerland). The pH of the feed solution was kept at 3.5 using sulfuric acid solution. Sample volumes of 6 mL were taken from the absorption tank at times of 1, 2, 3, 5, 7, 10, 15, 20, 25, 30, 45 and 60 min, in order to determine the cyanide concentration. Measurements of cyanide concentration using an automatic titration unit with AgNO_3_ 1 M and potentiometric end-point (Methron, Model 888 Tritando, Herisau, Switzerland) were carried out. To estimate the accountability of each test (overall mass balance of cyanide), a final sample from the feed tank was taken to quantify the cyanide concentration. In order to measure the water transferred between both phases during the assays, the absorption tank weight was monitored by using a digital scale (Boeco, Model BWL 61, Hamburg, Germany), in order to quantify the water transferred by osmotic effect from the feed solution. The water transfer occurring during the process operation was included in the cyanide mass balance to correct the cyanide concentration measured for each sample. The isothermal condition of the system was regulated by recirculating water at 15 °C through the tank jackets from a circulated thermo-regulated bath (JEIO Tech, Model RWE-2025, Daejeon, Korea). The flows of each phase were measured by a portable ultrasonic flowmeter (Cole-Palmer, Model 32615-68, Chicago, IL, USA).

Each test was run at three different cyanide solution flow rates ranging from 355 to 593 mL/min). A feed flow/absorption flow ratio of 1.0 was kept constant for all the experiments. Each test condition was carried out in duplicate. The cyanide recovery was estimated using a cyanide mass balance each time, according to the following equation:

(1) $$\mathrm{RecCN}=\frac{M_{\mathrm{CN}}^{0}{-M}_{\mathrm{CN}}^{t}}{M_{\mathrm{CN}}^{0}}\cdot 100$$

where RecCN is the cyanide recovery at each time (%), $M_{\mathrm{CN}}^{0}$ is the cyanide mass in the feed solution at time 0 (in g), and $M_{\mathrm{CN}}^{t}$ is the cyanide mass in the feed solution at each measured time (g). This last parameter was estimated using the cyanide concentration measured in the absorption tank and a mass balance that measured the total mass contained in the absorption tank at each time.

### 2.2. Hollow-Fiber Membrane Contactor (HFMC) Modules

**Figure 2:**
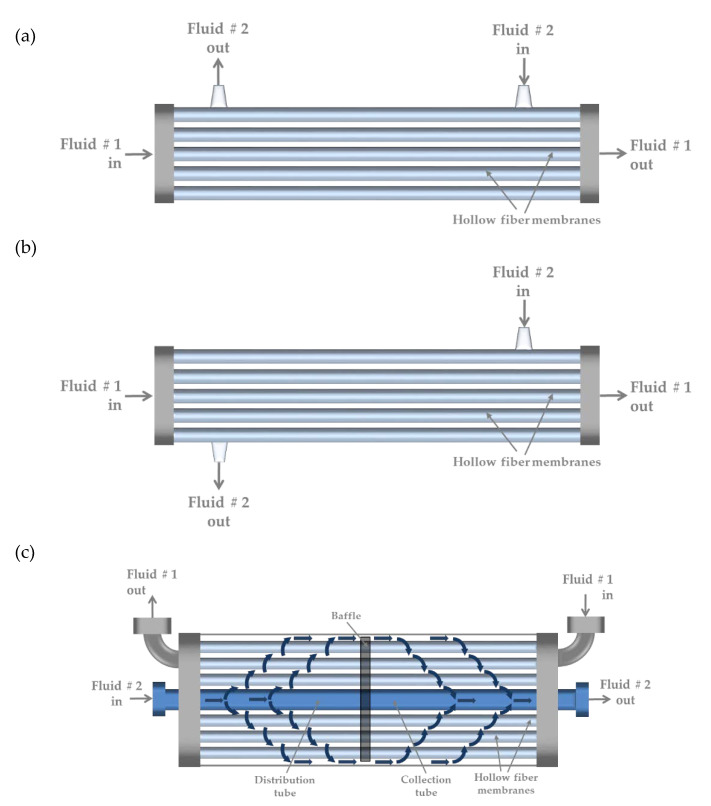
Schemes of the hollow-fiber membrane contactor (HFMC) modules (3M-LiquiCel^TM^) assayed in this study. (**a**) 1.7 × 5.5 Mini Module. (**b**) 1.7 × 10 Mini Module. (**c**) 2.5 × 8 Extra Flow Module. Fluid 1 and Fluid 2 correspond to the absorption and feed solution, respectively.

For each experimental set-up, three different HFMC modules from 3M-LiquiCel^TM^ (3M, Charlotte, NC, USA) were used: 1.7 × 5.5 Mini Module (Figure 2a), 1.7 × 10 Mini Module (Figure 2b), and 2.5 × 8 Extra Flow (Figure 2c). These HFMC modules differ among themselves in the mass-transfer area and the hydrodynamic configuration of the shell side. Both characteristics should determine the module performance from waste/process streams. For each HFMC module, each test was performed at three different feed flows: 356, 469, 591 mL/min for the 1.7 × 5.5 Mini Module; 355, 479, 588 mL/min for the 1.7 × 10 Mini Module; and 356, 552, 592 mL/min for the 2.5 × 8 Extra Flow. Table 1 shows the main characteristics of each HFMC module used in this study.

The experiences of cyanide recovery for each studied HFMC module were performed in duplicate. The comparison of recovery performance for each flowrate were carried out by an analysis of variance (ANOVA) test (α = 0.05) using Excel 2016 software (Microsoft, Redmond, WA, USA, 2016).

### 2.3. Determination of Mass-Transfer Correlations

Using the results of the cyanide concentration with respect to the process time for each feed flow condition, and applying an overall mass balance to the system (Figure 1), the overall mass-transfer coefficient was determined by Equation [15]:

(2) $$K=\frac{V}{A\;t}\ln\left(\frac{{\left[\mathrm{HCN}\right]}_{0}}{\left[\mathrm{HCN}\right]}\right)$$

where K is the overall mass-transfer coefficient (m/s), V is the total volume of the feed tank (m^3^), A is the total contact area of the HFMC module (m^2^), t is time (s), [HCN]_0_ is the initial hydrogen cyanide concentration in the feed tank (kmol/m^3^), and [HCN] is the hydrogen cyanide concentration in the feed tank at time t (kmol/m^3^). In this work, the Equation (1) was linearized to graph a logarithm of the HCN concentration (ln([HCN]_0_/[HCN])) with respect to the time. From the slope of this curve, the K value was obtained. Equally, the local mass-transfer coefficient was determined using Equation [8]:

(3) $$\frac{1}{K}=\frac{1}{k_{L}}+\frac{d_{\mathrm{in}}}{m_{\mathrm{HCN}}{\;k}_{m}{\;d}_{\mathrm{ml}}}$$

where k_L_ is the local mass-transfer coefficient in the boundary layer of the feed cyanide solution circulating by the shell side of the HFMC module (m/s), d_in_ is the inner diameter of the fibers (m), m_HCN_ is the partition constant of HCN in the gas phase per mole of HCN in the liquid phase (kmol/kmol), which represents the liquid feed-gas equilibrium described by Henry’s law for HCN, k_m_ is the local mass-transfer coefficient through the gas phase in the membrane pores (m/s), and d_ml_ is the logarithmic mean diameter of the fibers (m). The values of k_m_, m_HCN_ and d_ml_ were estimated following the methodology described by Estay et al. (2013) [8]. The membrane resistance could be neglected since the phase of cyanide solution represents more than 99% of the mass-transfer resistance [8]. The local mass-transfer coefficient in the shell side, k_L_, is related with the Sh number as follows:

(4) $$\mathrm{Sh}=\frac{k_{L}d_{e}}{D_{\mathrm{HCN}}}$$

where d_e_ is the characteristic length represented here by the equivalent diameter of the shell side (m), and D_HCN_ is the diffusion coefficient of HCN in water (m^2^/s). A typical mass-transfer correlation for determining the Sh number can be described as follows [15]:

(5) Sh=α Reβ Sc0.33

where Re is the Reynolds number, Sc is the Schmidt number, and α and β are parameters depending on the system hydrodynamic. Equation (5) can be linearized using natural logarithms to obtain the Sh number values with respect to the Re number. This last one is expressed as follows:

(6) Re=deρvμ

where ρ is the cyanide solution density (kg/m^3^), µ is the cyanide solution viscosity (Pa∙s), and v is the velocity of the cyanide solution flow in the shell side (m/s). The equivalent diameter depends on the HFMC module configuration in the shell side. This diameter and the respective velocity in the shell side of each HFMC module were estimated by using the equations described in Table 2.

## 3. Results and Discussion

### 3.1. Cyanide Recovery

**Figure 3:**
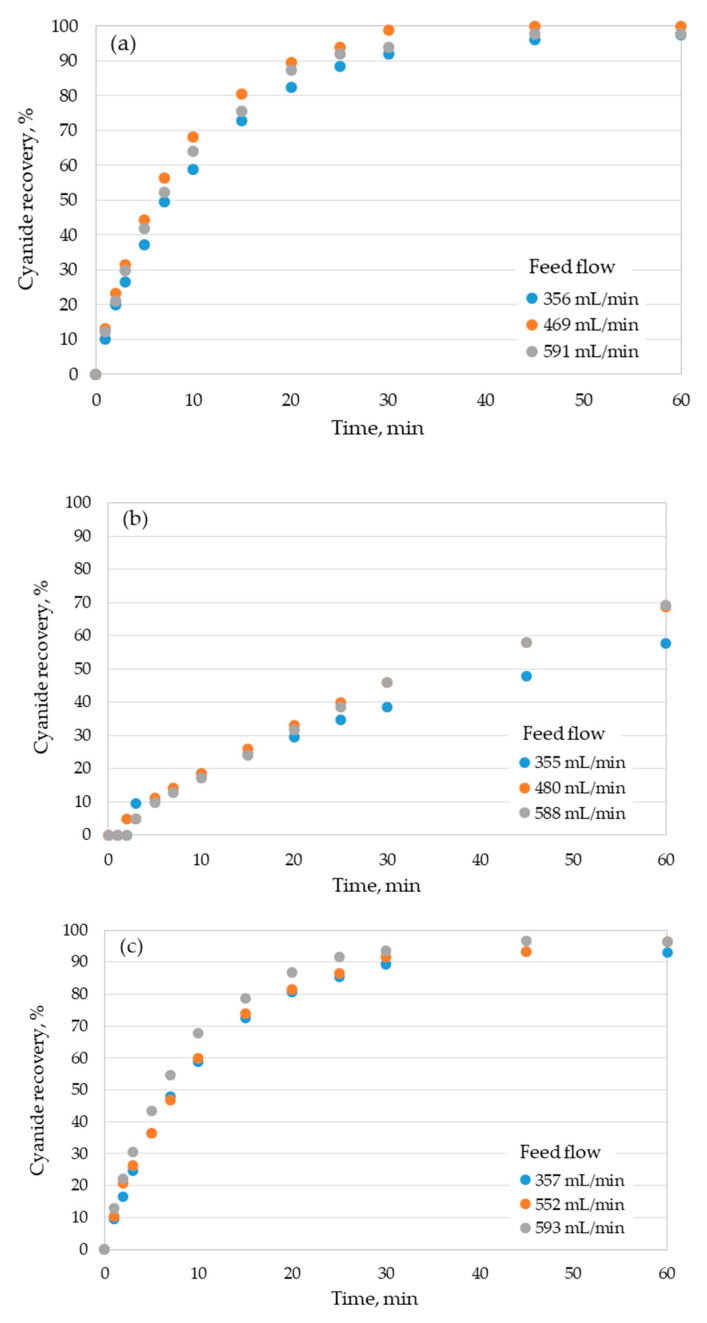
Cyanide recovery with the process time for each HFMC module operated at different feed flows and pH 3.5. (**a**) 1.7 × 5.5 Mini Module, (**b**) 1.7 × 10 Mini Module, and (**c**) 2.5 × 8 Extra Flow.

According to the operating conditions and type of module studied, different performances of cyanide recovery were obtained (Figure 3).

In general, for the 1.7 × 5.5 Mini Module and 2.5 × 8 Extra Flow modules, cyanide recoveries higher than 96% were reached at process times of 60 min, independent of the feed flow (Figure 3a,c). The only exception was cyanide recovery at the lowest feed flow operated in the 2.5 × 8 Extra Flow module, which had a value of 93%. By contrast, the maximum cyanide recovery achieved in the 1.7 × 10 Mini Module was around 70% at 60 min for the two higher feed flows assessed (480 and 588 mL/min) (Figure 3b), whereas a recovery of ~60% at a feed flow of 355 mL/min was obtained. From these results, we can assert that the 1.7 × 5.5 Mini Module and 2.5 × 8 Extra Flow modules are very attractive alternatives, since in both systems cyanide recoveries higher than 90% can be achieved at 30 min of operation for all feed flows tested. The statistical analysis showed that there are not significant differences in the cyanide recovery between the modules 1.7 × 5.5 Mini Module and 2.5 × 8 Extra Flow modules for the feed flowrates studied 356–357 mL/min (F-ratio = 0.4856; p = 0.7462), 469–552 mL/min (F-ratio = 0.1044; p = 0.9805), 591–593 mL/min (F-ratio = 0.0226; p = 0.9953). On the other hand, the 1.7 × 10 Mini Module showed significant differences (p ≤ 0.05) in the cyanide recovery with the other studied modules.

**Figure 4:**
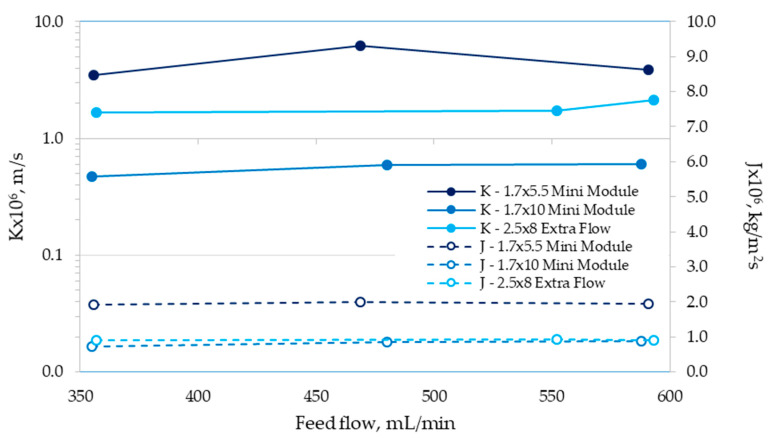
Overall mass-transfer coefficient (K) and flux of HCN at 60 min (J) for each HFMC module operated at different feed flows.

Surprisingly, we did not observe an expected direct relationship between the cyanide recovery performance and the mass-transfer area of the modules. In fact, the mass-transfer area of the 1.7 × 10 Mini Module is 44% larger than the 1.7 × 5.5 Mini Module (0.5 vs. 0.8 m^2^, Table 1), although the second one allows cyanide recoveries at least 28% higher than the 1.7 × 10 Mini Module to be reached (Figure 3a,b). The differences found between both modules seemed to be caused by a difference in the overall mass-transfer coefficients, since both systems possess an identical driven force of the process (cyanide concentration gradient). In addition, the 2.5 × 8 Extra Flow module has a mass-transfer area of at least 43% higher than the 1.7 × 5.5 Mini Module (1.4 vs. 0.8 m^2^, Table 1), but both systems showed very similar cyanide recovery results (Figure 3a,c). In order to clarify the relative importance of mass-transfer coefficient in the behaviors found, the overall mass-transfer coefficients for each module with respect to the feed flow were estimated using Equation (2). These results are shown in Figure 4, where the values obtained for the flux of HCN (mass of transferred HCN per time and mass-transfer area, J, kg/m^2^s) are also shown.

Results shown in Figure 4 indicate that the 1.7 × 5.5 Mini Module reached the higher values for K, ranging from 3 × 10^−6^ to 6 × 10^−6^ m/s, whereas the J values reached around 2 × 10^−6^ kg/m^2^s, in comparison with the other modules assessed. From the values obtained for the overall mass-transfer coefficient, it is possible to explain the higher cyanide recoveries obtained by the 1.7 × 5.5 Mini Module compared to those found for the 1.7 × 10 Mini Module, although this last one has a higher mass-transfer area. Alternatively, the K values for the 2.5 × 8 Extra Flow fluctuated between 1.6 × 10^−6^ and 2 × 10^−6^ m/s, which mean almost 50% lower values than the 1.7 × 5.5 Mini Module. These discrepancies in the K values compensate for the existing differences in the mass-transfer areas of both modules, which determined the similar cyanide recoveries reached for each feed flow (Figure 3). The higher overall mass-transfer coefficients achieved for the 1.7 × 5.5 Mini Module also explain the results obtained for the flux of HCN (Figure 4), in this case one order of magnitude higher than the 1.7 × 10 Mini Module and the 2.5 × 8 Extra Flow. The larger area of mass transfer for these two modules also determines a lower flux of HCN. On the other hand, the Flux values for the 1.7 × 10 Mini Module and 2.5 × 8 Extra Flow module were similar (Figure 4), although the mass-transfer area for the last one case was at least 40% higher (Table 1) than the 1.7 × 10 Mini Module, and the K value reached was higher as well. This effect is explained because of the cyanide concentration gradient of the 1.7 × 10 Mini Module at 60 min is higher than the value obtained by the 2.5 × 8 Extra Flow module, due to the lower cyanide recovery achieved by the 1.7 × 10 Mini Module. When the Fluxes are compared at the same value of cyanide recovery (between 60 and 70%), the 2.5 × 8 Extra Flow module achieved values between 2.8 × 10^−6^ and 3.8 × 10^−6^ kg/m^2^s (up to 78% higher than the 1.7 × 10 Mini Module). At the same cyanide recovery, the 1.7 × 5.5 Mini Module reached Flux values ranging from 7 × 10^−6^ to 8 × 10^−6^ kg/m^2^s.

The better performance of cyanide recovery observed for the 1.7 × 5.5 Mini Module can be explained by the location of the inlet and outlet flows in the shell side (Figure 2a), which promotes axial flows with respect to fibers, increasing the turbulence and thereby the mass-transfer coefficient in this side of the module [11,16]. By contrast, for the 1.7 × 10 Mini Module, the location of the inlet and outlet flows into the shell side promote a parallel flow with respect to the lumen side (Figure 2b). This configuration limits the increase in turbulence in the system and, consequently, in the mass-transfer coefficient of the shell side. In addition, this configuration could promote dead zones in the corners of the inlet and the outlet. Furthermore, the relationship L/d_e_ is of 170 for the 1.7 × 5.5 Mini Module, and 223 for the 1.7 × 10 Mini Module. The lower relationship L/d_e_ found in the 1.7 × 5.5 Mini Module allows limiting the development of a parallel flow through the shell side, enhancing the cyanide recovery performance.

Previous results [8] obtained for K and J values in the 1.7 × 5.5 Mini Module are in agreement with the results reported in this study (Figure 4). Estay and colleagues [8] reported K values ranging between 3 × 10^−6^ and 6.6 × 10^−6^ m/s for feed flows of 500 to 2,000 mL/min, and J values of around 1.2 × 10^−6^ kg/m^2^s.

**Figure 5:**
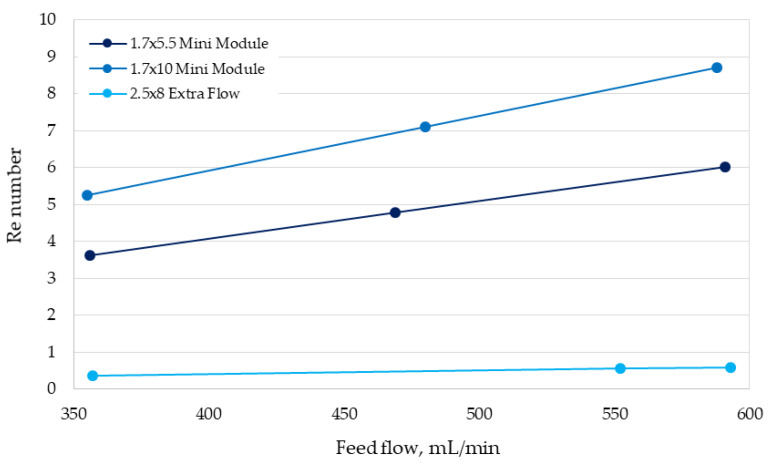
Values of the Reynolds number (Re) for each HFMC module with respect to the feed flow.

The results obtained for the 2.5 × 8 Extra Flow were unexpected, since the center tube and center baffle incorporated in the shell side in theory promote axial flows, which it should increase the mass-transfer coefficient in this side of the module. Moreover, the larger mass-transfer area of this module should enhance the cyanide recovery, a fact that is not in agreement with results shown in Figure 3c. However, a recent study reported by Pozzobon and Perré (2020) [16], who performed a continuous fluid dynamics (CFD) modeling, demonstrated that the turbulence in the shell side notably increases in the boundaries around the center baffle, but decrease in the extremes of the module, resulting in a similar behavior to the 1.7 × 10 Mini Module in a part of the shell side. In order to understand the fluid dynamics behavior for the modules assayed in our study in more depth, the Re numbers for each operational condition were estimated (Figure 5). It can be seen that the Re numbers obtained here for the 2.5 × 8 Extra Flow were very low (lower than 1.0) for the feed flows assessed, in comparison with the other conditions analyzed.

The low Re numbers achieved in the 2.5 × 8 Extra Flow are explained by the feed flows used in this study (350–600 mL/min), which are lower than the minimum flow recommended by the supplier module (1670 mL/min). In this work, feed flows, lower than those recommended for the 2.5 × 8 Extra Flow were chosen based on the comparison needed with the others modules tested here. Hence, the scaling-up study must consider the optimum operation range for each module; however, this selection must also consider the similarity in the Re number for all modules analyzed in order to scale-up using a pivotal operational parameter, just as the critical Re number does.

On the other hand, the good performance for cyanide recovery of the 1.7 × 5.5 Mini Module suggests that this type of configuration should be used to proceed with the scaling-up of the recovery process. However, previous results [11,12] show values of the mass-transfer coefficients for the Extra Flow configuration up to three orders of magnitude higher than those obtained by the 1.7 × 5.5 Mini Module. Hence, industrial scaling-up of an HFMC-based operation must consider in its selection process a module that can achieve the highest mass-transfer coefficients by increasing the system’s turbulence.

On the other hand, it should be pointed out that the effect of the studied feed flow rates on the cyanide recovery results for the 1.7 × 5.5 Mini Module (F-ratio = 0.2900; p = 0.9170), 1.7 × 10 Mini Module (F-ratio = 0.0836; p = 0.9990), and 2.8 × 8 Extra Flow (F-ratio = 0.1100; p = 0.9896) was found to be insignificant. The accountability of these tests ranged between 2% and 5%, thereby the experimental error of each result could vary around these values.

It is necessary to mention that the mass variations in the absorption tank were lower than 1% for all tests, which indicates a low water transference from the feed solution into the absorption tank or possible effects of pore wetting in the membrane.

### 3.2. Determination of Mass-Transfer Coefficients

The values of the overall mass-transfer coefficients estimated in this study using Equation (2) allowed the following to be obtained: the local mass-transfer coefficients in the shell side (Equation (3)), the Sh numbers (Equation (4)), and the parameters α and *β* (Equation (5)) for all HFMC modules operated under different feed flows. In Table 3 the mass-transfer correlations are presented.

The mass-transfer correlations for the shell side obtained here were compared with those used and validated in previous works [8,10,11] using the 1.7 × 5.5 Mini Module, and based on the correlation by Basu et al. [17,18] were described as:

(7) Sh=17.4(1−ϕ) deLRe0.6Sc0.33

where *ϕ* is the packing factor of the module. The values obtained from Equation (7) for the same feed flows assessed in this work were slightly lower than the results obtained using the correlations shown in Table 3, reaching values of the local mass-transfer coefficient ranging between 2.35 × 10^−6^ and 3.86 × 10^−6^ m/s. However, Basu et al.’s correlation has a higher exponent for the Re number (0.6) with respect to the correlation determined here (0.15). Hence, for feed flows higher than 600 mL/min, Basu et al.’s correlation will predict higher mass-transfer coefficients than those determined in this work. According to Basu et al. [17,18], their correlation is valid for Re number from 3 to 60, a wider range than the one shown in Table 3. In this context, both correlations can be used, but they are limited to the validity range of the Re number defined.

In the case of the 1.7 × 10 Mini Module, there is no background for mass-transfer correlations, thereby the correlation obtained here is a good approach for low Re numbers.

For the case of the 2.5 × 8 Extra Flow module, Table 4 shows the mass-transfer correlations used in this work, which are based on previous evidence reported by other authors for different systems [11].

**Figure 6:**
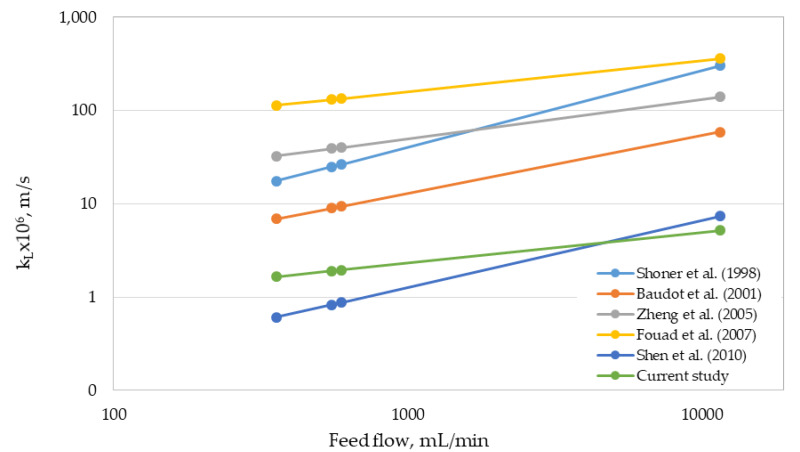
Comparison of local mass-transfer coefficients for the 2.5 × 8 Extra Flow module estimated from different correlations with respect to the feed flow.

For the 2.5 × 8 Extra Flow, the local mass-transfer coefficient (k_L_) of the shell side was estimated using the mass-transfer correlations listed in Table 4 and the correlation determined in this study. This estimation was performed for the range of feed flows assessed and an extrapolated value closer to the maximum flow recommended by the supplier (Table 1). Figure 6 shows the results of this simulation.

The correlation determined in this work predicted lower local mass-transfer coefficients in comparison with the other correlations analyzed, except for the correlation by Shen et al. [23]. However, for the extrapolated feed flow, our correlation predicted the lowest value of the local mass-transfer coefficient. The slope of our correlation is lower than those calculated for the rest of the correlations investigated (see Figure 6). Unfortunately, and as previously described, the Re ranges where the 2.5 × 8 Extra Flow module was tested were very low. For this reason, an extrapolation of more than 20 times the maximum flow assessed is very risky for scaling-up purposes. Moreover, the wide range of values for the mass-transfer coefficients obtained from different correlations (that in some cases results in 2 orders of magnitude) forces the scaling-up methodology for this type of modules to be tested under real operational conditions, using a Re number similar to that expected at industrial scale. As a result, further studies must be focused on testing a wide range of feed flows, assuring high values of Re number.

## 4. Conclusions

Considering a future industrial application in gold mining, an experimental comparison of the performance of three different HFMC modules in recovering cyanide from aqueous solutions was conducted. The modules tested had different configurations in terms of feeding flows and mass-transfer area. Results were compared through mass-transfer analysis, showing that the 1.7 × 5.5 Mini Module—with smaller mass-transfer area—had a similar cyanide recovery performance to those found for the larger area module, the 2.5 × 8 Extra Flow. Both modules reached cyanide recoveries over 93% at 60 min operation for all flow rates tested. Moreover, the HFMC module with medium mass-transfer area, 1.7 × 10 Mini Module, achieved cyanide recovery values lower than 70% at 60 min operation. These results can be explained by the differences in the mass-transfer coefficients determined for each HFMC module. The 1.7 × 5.5 Mini Module reached the highest values of mass-transfer coefficient, which was explained by its configuration that promotes the axial flow through the fibers at the inlet and outlet of the module shell side. By contrast, the 1.7 × 10 Mini Module possesses a configuration with parallel flows through the fibers and the 2.5 × 8 Extra Flow module requires higher feed flows to increase the Re values. In addition, distinct mass-transfer correlations for each module were determined, which can be used at the ranges of Re values assayed. The performance assessment at laboratory scale for different module configurations could allow implementing an affordable methodology to easily scale-up laboratory results to pilot plant or industrial scale. However, the results obtained here demonstrate the relevance of the feeding flows definition in which similar Re values are determined for all modules.

## Figures and Tables

**Table 1:** Characteristics of the HFMC modules [14].

Description	Unit	1.7 × 5.5 Mini Module	1.7 × 10 Mini Module	2.5 × 8 Extra Flow
Feed flow capacity	m³/h	<0.15	<0.12	0.1–0.7
Membrane material	-	Polypropylene	Polypropylene	Polypropylene
Fiber outer diameter	µm	300	300	300
Fiber inner diameter	µm	220	220	220
Fiber length	m	0.12	0.25	0.15
Shell inner diameter	m	0.043	0.045	0.056
Membrane porosity	-	0.5	0.4	0.5
Mass-transfer area	m²	0.5^1^	0.8^1^	1.4^2^
Centre tube diameter	m	-	-	0.0222

^1^ Area based on the inner diameter of the fibers. ^2^ Area based on the outer diameter of the fibers.

**Table 2:** Equivalent diameter and velocity equations of the shell side for each HFMC module [11].

Module		Equivalent Diameter	Velocity
1.7 × 5.5 Mini Module		$d_{e}=\frac{d_{s}^{2}{-\mathrm{nd}}_{\mathrm{out}}^{2}}{d_{s}{+\mathrm{nd}}_{\mathrm{out}}}$	$v=\frac{Q_{s}}{A_{s}};A_{s}{=(d}_{s}^{2}{-\mathrm{nd}}_{\mathrm{out}}^{2})\frac{\pi }{4}$
1.7 × 10 Mini Module		$d_{e}=\frac{d_{s}^{2}{-\mathrm{nd}}_{\mathrm{out}}^{2}}{d_{s}{+\mathrm{nd}}_{\mathrm{out}}}$	$v=\frac{Q_{s}}{A_{s}};A_{s}{=(d}_{s}^{2}{-\mathrm{nd}}_{\mathrm{out}}^{2})\frac{\pi }{4}$
2.5 × 8 Extra Flow		$d_{e}=\frac{d_{s}^{2}{-d}_{\mathrm{ct}}^{2}{-\mathrm{nd}}_{\mathrm{out}}^{2}}{{\mathrm{nd}}_{\mathrm{out}}}$	$v=\frac{{2\;Q}_{s}\ln\left(\frac{d_{s}}{d_{\mathrm{ct}}}\right)}{{\pi L(d}_{s}{-d}_{\mathrm{ct}})}$
Nomenclature:			
d_s_	: inner diameter of the shell (m)		
d_out_	: outer diameter of the fibers (m)		
d_ct_	: outer diameter of the center tube in the Extra-Flow module (m)		
n	: number of fibers contained in the HFMC module		
Q_s_	: cyanide solution flow fed into the shell side (m^3^/s)		
A_s_	: cross-sectional area of the flow in the shell side (m^2^)		
L	: length of the fibers (m)		

**Table 3:** Mass-transfer correlations obtained in this study for each HFMC module, under the validity range of the Re number.

Module	Mass-Transfer Correlation	Validity Range Based on the Re Number
1.7 × 5.5 Mini Module	Sh=0.1514Re0.2603Sc0.33	3.5–6.0
1.7 × 10 Mini Module	Sh=0.0171Re0.5Sc0.33	5.0–9.0
2.5 × 8 Extra Flow	Sh=0.0994Re0.3303Sc0.33	0.3–0.6

**Table 4:** Mass-transfer correlations reported in previous studies for the 2.5 × 8 Extra Flow.

Module	Mass-Transfer Correlation	Validity Range Based on the Re Number
Shoner et al. [19]	Sh=1.76Re0.82Sc0.33	0.02–2.0
Baudot et al. [20]	Sh=0.56Re0.62Sc0.33	3–30
Zheng et al. [21]	Sh=2.15Re0.42Sc0.33	0–20
Fouad et al. [22]	Sh=6.8695Re0.33344Sc0.33	0–0.1
Shen et al. [23]	Sh=0.055Re0.72Sc0.33	0.1–250
